# Sub capsular splenectomy for delayed spontaneous splenic rupture in a case of sickle cell anemia

**DOI:** 10.1186/1749-7922-4-17

**Published:** 2009-05-06

**Authors:** Dhananjaya Sharma

**Affiliations:** 1 Department of Surgery, Government NSCB Medical College, Jabalpur (MP) 482 003, INDIA

## Abstract

Splenic ruptures are mostly due to trauma and manifest at the moment of injury with symptoms of acute intraperitoneal hemorrhage and shock. Spontaneous/pathological and delayed rupture of the spleen is not unknown. A case of delayed spontaneous splenic rupture in a case of sickle cell anemia is being reported, which was treated with sub capsular splenectomy (from within the pseudo capsule formed due to inflammation).

## Background

Many pathological conditions of spleen predispose it to spontaneous rupture, diagnosis of which can be delayed due to its unusual presentation. Splenectomy is often required for splenic rupture, both for its acute and chronic presentations. Chronic splenic rupture may be associated with dense peri splenic adhesions making this surgery a difficult one. In such a scenario, avoidance of iatrogenic trauma to neighboring organs is of paramount importance. Sub capsular Splenectomy (from within the pseudo capsule formed due to inflammation) is an alternative technique and allows a safe splenectomy in cases having dense peri splenic adhesions.

## Case report

**Figure 1:**
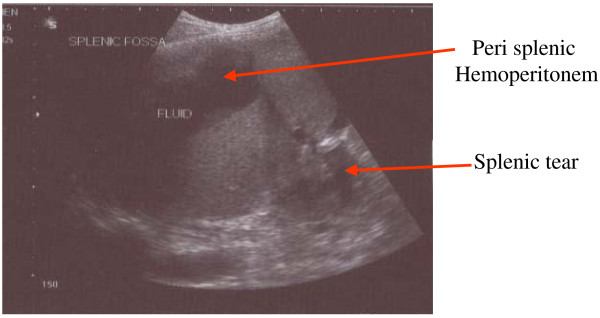
**Chronic rupture of spleen with hemoperitonem in perisplenic area**.

**Figure 2:**
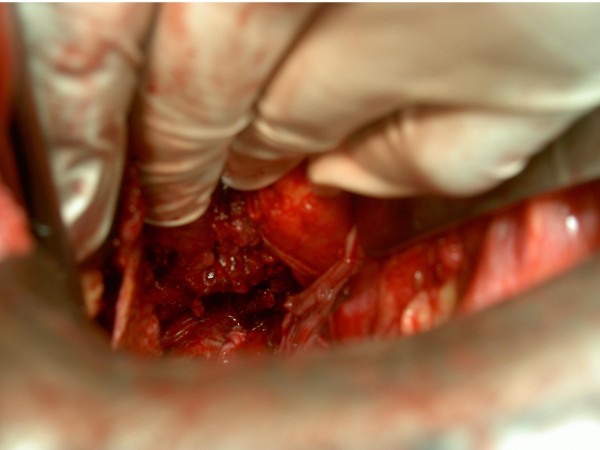
**Sub capsular splenectomy being performed**.

**Figure 3:**
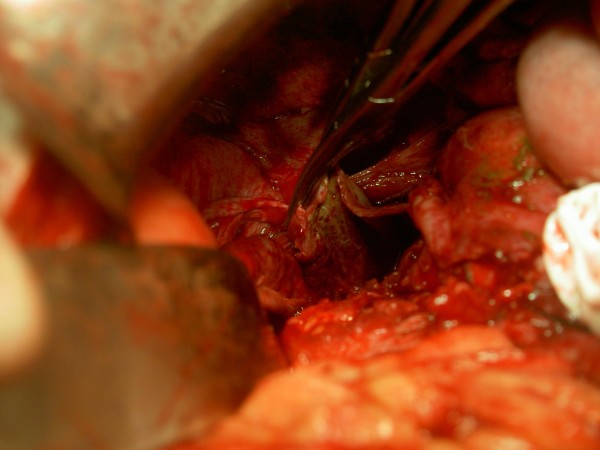
**Thickened and densely adherent splenic capsule**.

**Figure 4:**
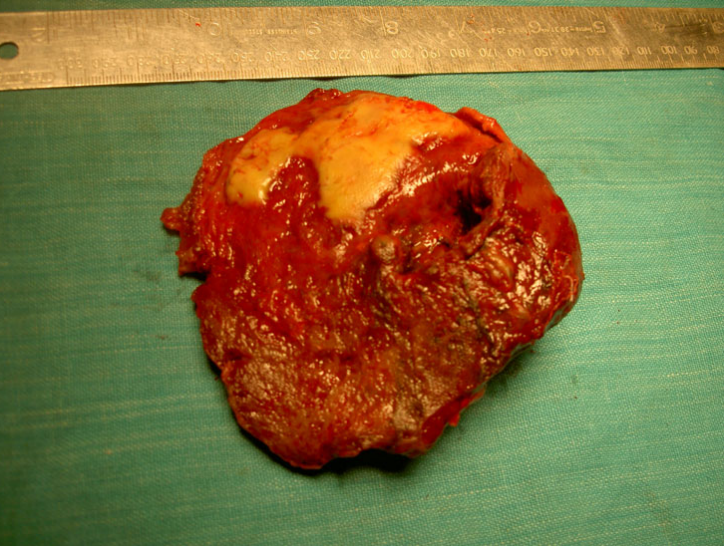
**Removed spleen**.

KSM, a 50 year old man presented with severe pain over left hypochondrium and left lower chest wall, moderate fever on and off for one month. Pain increased on deep inspiration and radiated to left shoulder. There was no history of trauma or any disease process. On examination, only positive sign was some tenderness over left hypochondrium. Ultrasonography revealed chronic rupture of spleen with some hemoperitonem in the perisplenic area and small pleural effusion. (Figure 1) Biochemical workup did not show any abnormality, except a positive test for sickle cell trait. Patient was taken up for splenectomy because of severe pain. On exploratory laparotomy left quadrant was found cordoned off by omental adhesions. On taking down the adhesions, 250 ml of darkish blood was drained form the area around the spleen. Dense adhesions prevented separation of spleen from diaphragm, left lobe of liver, stomach and left flexure of colon. Attempt was made to ligate splenic vessels, by opening the lesser sac, but dense adhesions prevented success of this step. Sub capsular splenectomy (SCS, from within the pseudo capsule formed due to inflammation) starting from near the diaphragm, was performed so as to avoid inadvertent iatrogenic trauma to neighboring structures. (Figure 2) Splenic vessels were identified inside the capsule and ligated by transfixing en-mass with 1-0 silk. Splenic capsule was found thickened and densely adherent to neighboring structures. (Figure 3) Abdomen was closed after a thorough lavage and a tube drain was inserted in the left sub diaphragmatic region. Removed spleen (Figure 4) was sent for histopatholgical examination.

There was 300 ml of sero-sanguinous fluid in the drain on first post operative day, which gradually subsided and drain could be removed on fourth post operative day. Patient made an uneventful recovery.

## Discussion

Causes of pathological rupture of the spleen can be classified as (1) Infections e.g., viral (infectious mononucleosis), parasitic (malaria, dengue), bacterial (abscess); (2) Congenital (cyst); (3) Metabolic (Gaucher's disease); (4) Degenerative (Amyloidosis). (5) Hematological Malignancy (leukemia, lymphoma), (6) Vascular (rupture of intrasplenic aneurysm, coagulopathy or infarct), (7) Secondary to chronic pancreatitis, and (8) Miscellaneous causes like Sickle cell disease, Peliosis, cytoreductive chemotherapy etc [1-6]. Various mechanisms of rupture of diseased spleen have been postulated: (1) Mechanical effect of distension secondary to disease infiltration of the spleen, especially the capsule; (2) Splenic infarct with capsular hemorrhage and subsequent rupture; (3) Defects in blood coagulation. Rupture probably results from a combination of these mechanisms rather than from any single mechanism [1]. In the present case there was no history of any event triggering splenic rupture, however, Sickle cell anemia is known to cause congestive splenomegaly, making it more prone to rupture [7].

Most patients thought to have delayed rupture of the spleen have, instead, delayed recognition of splenic rupture [8]. Lesser trauma resulting from minor falls or fights, often forgotten or unnoticed, is more likely to lead to delayed, so called spontaneous rupture. Subcapsular hematoma is the most common etiology for delayed splenic rupture [9]. But, Subcapsular Hematoma is neither a predictor for delayed splenic rupture, nor by itself an indication for operative management of the injured spleen in a hemodynamically stable patient [10]. Decision to operate must be taken based on imaging by ultrasonography or CT scan. The ultrasonologist was able to diagnose chronic rupture of spleen due to the presence of 'old' blood along with splenic rupture [11]. In the present case the decision to perform Splenectomy was taken due to severe pain.

Sub capsular nephrectomy is performed in cases of pyonephrosis with non-functioning kidney as tissue planes around the kidney are lost due to infective pathology. Presence of blood around spleen for one month may have led to dense perisplenic adhesions, which prompted the performance of SCS (from within the pseudo capsule formed due to inflammation), which led to safe and successful outcome in this case.

## Conclusion

Sub capsular Splenectomy (from within the pseudo capsule formed due to inflammation) is an alternative technique and allows a safe splenectomy in cases having dense peri splenic adhesions. This procedure avoids potentially dangerous attempts at removing all the dense adhesions and fibrin layer that might in some cases have formed a pseudo capsule. The knowledge of this procedure will be an additional weapon in the armamentarium of surgeons, when facing similar problem.

## Competing interests

The author declares that they have no competing interests.

## Consent

Written informed consent was obtained from the patient for publication of this case report and accompanying images. A copy of the written consent is available for review by the Editor-in-Chief of this journal.
